# Unicompartmental Knee Arthroplasty Is Cost-Effective in an Outpatient Setting

**DOI:** 10.7759/cureus.35059

**Published:** 2023-02-16

**Authors:** Nicholas F Cozzarelli, Andrew S Longenecker, Alex Uhr, Daniel E Davis, Jess H Lonner

**Affiliations:** 1 Department of Orthopaedic Surgery, Rothman Orthopaedic Institute at Thomas Jefferson University, Philadelphia, USA

**Keywords:** cost, ambulatory surgery center, outpatient, uka, unicompartmental knee arthroplasty

## Abstract

Introduction:Increasingly, unicompartmental knee arthroplasty (UKA) is being performed on an outpatient basis, with the growing utilization of ambulatory surgery centers (ASCs). The purpose of this study was to compare the costs of UKAs performed in an ASC to UKAs done in a hospital, either on an outpatient or inpatient basis.

Methods:This study involved three matched groups, each with 50 consecutive patients, undergoing UKA either on an outpatient basis in an ASC or a community hospital, or who were admitted overnight to the same community hospital. Identical perioperative analgesic regimens and care protocols were used in each group. The primary outcomes evaluated included direct facility costs. Secondary outcomes were postoperative complications and readmissions.

Results:Average age, gender ratio, and comorbidities were similar in all three cohorts. Only two patients in the study experienced complications and these were without secondary adverse consequences. Mean costs were substantially reduced when UKAs were performed in an ASC ($9,025) compared to a community hospital on either an outpatient ($12,032) or inpatient basis ($14,542).

Conclusion:UKA can be safely performed in the outpatient setting, in appropriately selected patients, at substantial cost savings, particularly when performed in an ASC.

## Introduction

The percentage of unicompartmental knee arthroplasties (UKAs) performed in the outpatient setting increased approximately fourfold from 2007 to 2015 [1]. According to the Centers for Medicare & Medicaid Services, the perioperative costs of knee arthroplasty in 2014 ranged from $16,504 to $33,072 [2]. If these costs associated with knee arthroplasty are left unchecked, hospitals and payers will be increasingly challenged.

Shift to ambulatory surgery centers

Strategies to improve quality and reduce costs are particularly germane, including risk stratification, perioperative medical optimization, reduced length of stay, avoidance of subacute rehabilitation and skilled nursing facilities, and transition of select cases to ambulatory surgery centers (ASCs) [3-5]. Medicare saved nearly $7 billion between 2007 and 2011, by shifting outpatient surgical procedures from hospitals to ASCs for patients considered low risk [6]. Similarly, a review of commercial medical claims data found that US healthcare costs are reduced by more than $38 billion per year due to the shift of outpatient cases to ASCs [7].

Outpatient total joint arthroplasty

Outpatient total knee arthroplasty (TKA) and total hip arthroplasty (THA) can be done safely and cost-effectively in appropriately selected patients [8-10] with surgical approaches that focus on decreasing soft tissue damage, improvements in perioperative anesthesia and analgesia, and institution of early rehabilitation and mobilization regimens [11,12]. For instance, Lovald et al. reported that the cumulative mean costs over a two-year postoperative period are $8527 less in TKA patients discharged from the hospital on the day of surgery [13].

Still, some are reluctant to perform TKA in an ASC. Generally, UKAs have a significantly lower risk of complications and readmissions than TKAs [14-16], which may lead more surgeons to consider performing UKAs in an ASC. When considering direct and indirect costs, UKA is more cost-efficient than TKA in any setting [17]. Although Medicare reimbursement is equal between outpatient TKA and UKA, total procedure cost, total supply cost, implant cost, and complication costs are lower in outpatient UKA compared to TKA [18].

Outpatient unicompartmental knee arthroplasty

While several studies have reviewed the safety and feasibility of outpatient UKA [19-21], there are little data on the potential cost savings incurred by shifting UKAs to the ASC setting. Ford et al. studied the safety and cost of outpatient UKAs in an ASC, but they did not compare these costs to UKAs performed in a hospital setting [22]. Additionally, Richter et al. compared the costs of UKA performed in the same hospital on an inpatient or outpatient basis; however, they did not analyze a group undergoing outpatient UKA at an ASC [23]. The direct costs of performing UKA in a general hospital on an inpatient basis have not been compared in detail to the costs of outpatient UKA performed in the same hospital or in a hospital-owned ASC.

The purpose of this study is to evaluate the direct costs and readmissions and complications of UKAs performed in an ASC compared to those done in a hospital both on an inpatient and outpatient basis. We hypothesize that the total direct costs of UKA will be lower in the outpatient setting compared to the total costs rendered in a hospital setting, whether inpatient or outpatient. We also hypothesize that there will be no difference in adverse events or readmissions between the three cohorts.

## Materials and methods

A retrospective review was performed of consecutive patients undergoing medial UKA between January 2012 and December 2014 at two facilities owned and operated by a single private health system - a general suburban community hospital and a suburban ASC. Neither the senior surgeon nor his practice or any of the co-authors had any ownership or financial relationship with the ASC or hospital. Institutional review board (IRB) approval was received prior to initiating the study.

Study cohorts

Consecutive cohorts of 50 patients in each of the three groups undergoing UKA were identified: UKA with one night stay in a community hospital (IP-CH); UKA on an outpatient basis in the same community hospital (OP-CH); and UKA in an ambulatory surgical center (OP-ASC).

The patients were studied during a transition from an inpatient to an outpatient stay at the particular health system. Before transitioning patients to the ASC, first a trial of 50 outpatient UKAs, in patients with an American Society of Anesthesiologists (ASA) score of 2 or less, was performed at the hospital to prove the safety, feasibility, and effectiveness of the perioperative protocols. Once confirmed, patients undergoing UKA with an ASA score of 2 or less were then transitioned to the hospital-owned ASC, and those initial 50 consecutive patients at the ASC were studied. A third cohort of 50 consecutive inpatient UKAs at the hospital before the transition period was retrospectively matched to the outpatient cohorts by age, ASA, and Charlson Comorbidity Indices (CCIs), to serve as controls. Thus, in this study, a group of 50 consecutive inpatient UKA patients was compared to the first 50 outpatient hospital UKA patients, and the initial 50 UKA patients at the ASC. Mean patient ages, gender distribution, CCIs, and body mass indices (BMIs) were comparable between groups (Table 1).

**Table 1:** Preoperative characteristics Values are given as mean ± SD or N (%). OP-ASC, outpatient-ambulatory surgery center; OP-CH, outpatient-community hospital; IP-CH, inpatient-community hospital; BMI, body mass index; CCI, Charlson Comorbidity Index.

	OP-ASC (n = 50)	OP-CH (n = 50)	IP-CH (n = 50)	P-value
Age (years)	58.2 ± 3.1	57.1 ± 3.0	59.2 ± 2.9	0.268
Gender				0.911
Female	30 (60)	30 (60)	28 (56)	
Male	20 (40)	20 (40)	22 (44)	
BMI (kg/m^2^)	27.9 ± 4.1	28.2 ± 3.2	28.1 ± 4.4	0.341
CCI	0.7 ± 1.2	0.8 ± 1.3	0.8 ± 1.3	0.583

Perioperative protocol

All operations were performed by a single fellowship-trained arthroplasty surgeon with extensive experience performing UKAs. In all three settings, the procedures were performed with the same UKA system, using identical standardized protocols throughout the care process. Since the hospital and the ASC contracts and management were under the same umbrella administration, implant costs were confirmed to be identical in each group.

Before surgery, all patients were pre-medicated with oxycodone 10 mg, acetaminophen 975 mg, and celecoxib 200 mg unless contraindicated due to allergies or other medical issues. All patients received spinal anesthesia with low-dose bupivacaine and pericapsular local infiltration of 30cc 0.5% bupivacaine and liposomal bupivacaine. In the post-anesthesia care unit (PACU), the pain was managed as needed with a combination of oral and intravenous pain medications. Postoperative nausea was managed with ondansetron as needed. Patients in the inpatient UKA group were all discharged on postoperative day one. All patients received the same postoperative physical therapy (PT) protocols and discharge pain medications.

Outcome measurements

Total direct facility costs of the procedure were recorded for each patient at each facility. This value represents costs associated with institution supplies and services, anesthesia services, implant selection, institutional implant costs, additional PACU medications and services required, and costs associated with operating room use, to measure the financial disparity between the two settings. We were unable to obtain individual lien item cost elements from each center due to institutional policies and contractual restrictions regarding the release of this otherwise proprietary information. All the patients were followed postoperatively for a minimum of 90 days by a nurse navigator to prospectively track and manage any postoperative complications or readmissions at different facilities. Any complications within 90 days resulting in emergency department (ED) visits or readmission were recorded.

The primary outcomes evaluated included total direct facility costs. Secondary outcomes included non-index hospital admissions (or readmission) and 90-day postoperative complications. A Kruskal-Wallis one-way analysis of variance test was conducted to compare the effects of surgical setting on costs related to UKA. P-values less than 0.05 were deemed statistically significant. All statistical analyses were performed using R Studio (version 3.6.3, Vienna, Austria).

## Results

Primary outcomes of interest

UKA in the OP-ASC group was the most cost-effective in comparison to the IP-CH and OP-CH groups (Table 2). The results of the testing reached statistical significance (p < 0.001), and the mean total direct cost was $9,025 for UKA in the OP-ASC, $12,032 for UKA in the OP-CH, and $14,542 for IP-CH.

**Table 2:** Comparison of total direct costs Data are represented as mean (range). Costs are given in US dollars. OP-ASC, outpatient-ambulatory surgery center; OP-CH, outpatient-community hospital; IP-CH, inpatient-community hospital.

	OP-ASC	OP-CH	IP-CH	P-value
Cost	$9,025 ($6,408-$11,108)	$12,032 ($7,108-$15,009)	$14,542 ($12,542-$17,635)	<0.001

Thus, the mean total direct cost of UKA in a hospital with an overnight stay was 20.86% greater than UKA performed at the same hospital on an outpatient basis and 61.13% greater than UKA performed in an ASC. The mean total direct cost of outpatient UKA in a general hospital was 33.32% greater than UKA performed in an ASC.

Secondary outcomes of interest

One complication occurred in a 49-year-old patient in the IP-CH group, who tripped downstairs nine days postoperatively, sustaining wound dehiscence requiring emergent irrigation and wound closure without adverse consequences. One patient in the OP-CH group required a return visit to the ED for bladder catheterization for urinary retention 10 hours post-operatively. None of the remaining 148 patients had adverse events, ED visits, or hospital readmissions in the 90-day postoperative period.

## Discussion

Shifting UKAs to an outpatient setting is cost-effective, on the basis of direct facility costs. While a study by Richter et al. found that UKA is more cost-effective on an outpatient basis in a hospital [23], our study also demonstrates significant further direct cost savings by shifting outpatient UKA cases from the hospital to the ASC. In our study, direct cost savings were 17% when shifting cases from an inpatient to an outpatient basis at the same general hospital and 38% when shifting to an ASC. Furthermore, performing an outpatient UKA at an ASC was 25% less costly than on an outpatient hospital basis. These cost savings are comparable to those observed when other common outpatient orthopedic surgical procedures were moved from a university hospital to a hospital-owned ASC, with savings ranging from 17% to 43%, primarily attributed to OR time and supply utilization [24].

Our study supports the safety and cost-effectiveness of UKA when perioperative protocols and expectations are managed in appropriately selected, risk-stratified patients, inasmuch as the incidence of complications and readmissions are low in UKA with same-day discharge, particularly when compared to TKA. Compared to commonly cited direct and indirect costs of TKA, our study shows that overall costs of care can be markedly lower in UKA [16,25,26]. For instance, Kremers et al. found that the mean direct hospital costs/care for 6,475 primary TKAs at one hospital was $15,673 (SD: $5,699) [27], and Weeks et al. found mean acute care costs in knee arthroplasty patients among multiple hospitals in 2013 ranged from $18,945 to $19,805 [28]. Chisari et al. found that overall facility costs of outpatient UKA were 13.51% less than outpatient TKA, despite equivalent reimbursement [18]. Furthermore, Bosch et al. found that despite there being a significantly lower risk for 90-day medical complications, reimbursement for outpatient UKA is decreased in comparison to inpatient UKA [29].

Understanding how costs accumulate is imperative to develop strategies to reduce these costs. Kremers et al. determined the components of direct medical costs in TKA and found that the largest proportion of hospitalization costs was for room and board (28%), operating room (22%), prostheses (13%), and physicians (16%) [27]. Shifting cases to the ASC can thus have a most profound impact on reducing facility costs.

Improvements in pain management and preoperative education, advanced arrangements for early outpatient post-operative PT, and standardized protocols have all played important roles in decreasing the duration of hospitalization and facilitated the transition of TKA and UKA to the outpatient setting [30]. Pain control has been shown to be a common reason for an overnight hospital stay after knee arthroplasty, therefore, optimizing safe opioid-sparing pain control is paramount. In our series, identical standardized analgesics were utilized across the three patient groups, and no patients had changes to the pain protocol or complications due to pain, indicating that pain control methods were both adequate and safe for facilitating same-day discharge after UKA.

Limitations

Potential limitations to this study are those inherent to a retrospective study without randomization. While the outpatient cases were done based on a prospective protocol change, shifting to outpatient surgery from an overnight admission, there was no formal randomization, which could potentially have a bearing on the cost. Nonetheless, the groups were well-matched by demographic variables, ASA, and comorbidities. Since our study looked only at lower-risk patients with an ASA score of 2 or less, the results may not be generalizable to patients with greater comorbidities. Regardless of the site, outpatient UKA was done safely and without a substantive risk of readmission after discharge. Additionally, more recent patients were not studied because subsequently, we have transitioned our UKAs to practice-owned ASCs, where we have more influence on cost structure and charges. While we were able to compare total direct costs at each site, we were unable to break down the costs of each individual lien item cost per site that contributed to the total costs of care, as this is proprietary information within this particular health system. We also did not factor in total costs for the entire 90-day episode of care or indirect costs. Nonetheless, we surmise that perioperative costs may otherwise be similar between groups, considering common protocols and limited complications. Finally, in this study, although a power analysis was not performed, since the primary outcome finding of the cost was significantly different, a post-hoc analysis was not required. Additionally, a power analysis was not performed for the secondary outcomes of complications and readmissions, such as in a study by Cody et al. [21].

## Conclusions

This study compared the direct costs of UKA performed on an inpatient basis to those incurred when UKA was performed on an outpatient basis in the same hospital, and at an ASC. The strength of this study is that it was performed within a single health system that controls charges and costs within both their general hospital and ASCs. Future studies should look at episode-of-care costs along with patient-reported outcomes in a larger retrospective study or a randomized control trial. We demonstrated that UKA can be safely performed in the outpatient setting in select patients. Further, there is a significant cost saving when UKA is performed in an outpatient setting and care is shifted from a general community hospital to an ASC.
